# Visual Size Matters: The Effect of Product Depiction Size on Calorie Estimates

**DOI:** 10.3390/ijerph182312392

**Published:** 2021-11-25

**Authors:** Aner Tal, Yaniv Gvili, Moty Amar

**Affiliations:** 1 School of Business, College of Law and Business, Ramat Gan 52573, Israel; anert@clb.ac.il; 2 School of Business Administration, Ono Academic College (OAC), Kiryat Ono 55000, Israel; mamar@ono.ac.il

**Keywords:** calories, visual cues, food judgment, biases, nutrition

## Abstract

Consumers’ calorie estimates are often biased and inaccurate. Even the presence of relevant nutritional information may not suffice to prevent consumer biases in calorie estimation. The current work demonstrates across two studies that visual cues given by larger product depictions lead to increased calorie estimates. Further, it demonstrates that these effects occur even when consumers are given, and notice, information about product quantity. The findings thus shed light on a novel biasing effect on consumer calorie evaluation, and, more generally, the findings provide evidence for the importance of visual inputs over textual ones in consumers’ nutritional assessment of food products. In this, the current research provides insights relevant to helping nutritional literacy via awareness of biasing influences on caloric assessment. In the same manner, the research also provides insights that may assist the regulator protecting consumers by highlighting factors biasing nutritional assessment.

## 1. Introduction

Policy regulations for food products focus on providing clear product information, with quantity and caloric information being two key elements. When provided to consumers, explicit nutritional information can alter their calorie estimation of food items purchased [1]. However, consumers make limited use of labels, and their effectiveness has been called into question [2,3,4,5]. When it comes to calorie assessment in particular, consumers may rely on other cues. Visual inputs, in particular, may offer a salient cue to guide calorie estimates and may override textual cues in guiding consumer judgment and decision making [6,7,8].

Caloric estimates in general tend to be problematic and open to distortion. Even given objective information, consumers may have difficulty estimating the calories in a package [9]. Specifically, quantities tend to be underestimated, which can lead to increases in caloric intake [10]. Namely, if consumers think a product is less caloric, they may feel legitimate in consuming more of it. Accordingly, biases in calorie estimates can affect eating behavior. For example, consumers may use overall the visual input provided by visual depiction of serving size on the package as a proxy for suggested serving size [11]. If they do lead to underestimation of calories, visual cues may lead to increased eating, and thus, potentially to overeating and obesity [12,13,14].

The general inaccuracy of consumers in calorie estimates leaves calorie estimates open to external influence and distortion. Consumers’ caloric estimates often rely on subjective interpretations of potentially unrelated cues [15,16]. A variety of factors, many of which are unrelated to actual quantity, can bias calorie estimates [17]. These distortions may in turn influence purchase decisions and consumption. In general, the more salient the cues, the more likely they are to influence calorie estimation [18,19]. Visual cues are particularly salient, leading them to be particularly important in guiding estimates [20,21].

In the current work, we examine how the size of visual cues such as product image can affect calorie estimation. Specifically, we show that larger product depictions lead to increased calorie estimation. We argue that this is due to the natural association between size, quantity, and calories. Further, we argue that given the salience of visual cues, the visual input provided by product image size may override objective nutritional information and alter calorie estimates even in the presence of information relevant to calorie assessment.

The remainder of this article is organized as follows. First, we briefly review the importance of quantity estimates in the context of consumption biases. We then discuss the general importance of imagery in consumer judgment and choice. Next, we explore how the size of pictures may affect quantity and calorie estimates, leading to formal hypotheses regarding our proposed bias. Two studies provide supporting evidence for our hypotheses, followed by a discussion of future research and practical implications.

## 2. Literature Review and Theoretical Development

### 2.1. Factors Affecting Calorie and Quantity Estimates 

Food quantity estimates have an important role in determining consumers’ food choices and consumption quantity [22,23]. Consumers may rely on their knowledge or estimates of the quantity or calories in a food product in deciding what and how much to eat. In general, perceiving food products to be less caloric, as well as lower quantity, leads to increased consumption [22,24]. Calorie estimates have been shown to be particularly influential in affecting food serving [25,26]. Accordingly, posting calorie values of food in restaurants has been shown to impact consumer choice [13,27,28].

Reduced estimations of quantity or calories may thus potentially lead to overconsumption, and consequently contribute to obesity [13,29,30]. Specifically, if consumers perceive a product as containing a lower quantity or fewer calories, they may eat more of it, as evidenced in research showing that serving food in small packages increases consumption [31,32]. Overall, consumers tend to underestimate calories, which can contribute to overeating [33]. Overeating, in turn, may lead to extensive adverse health effects and concomitant social costs [34,35,36]. Accordingly, understanding the factors biasing calorie estimation is an important area for research.

In general, consumers struggle with calorie estimates and are often woefully inaccurate [22,37]. Similarly, consumers are often inaccurate in assessing product quantity [10,15,33,38,39,40,41]. Notably, estimations of calories and quantity tend to be correlated, such that increased quantity and increased calories go hand in hand [42,43]. Quantity information should, in fact, affect calorie evaluations since calories are a function of quantity and caloric density. Remarkably, inaccuracies in quantity and calorie estimation may exist even when consumers are given objective nutritional information [42]. While such information can help improve accuracy, it is no guarantee of accurate assessment and the avoidance of biases [16,39,44,45].

A variety of factors have been implicated in calorie underestimation. Individual differences in gender, weight, and dieting status, for example, all influence the accuracy of calorie estimates [46,47]. Increased body mass also contributes to calorie underestimation [16,39,44,45]. Conversely, dietary restraint can improve accuracy in calorie estimation [16,23]. Finally, differences in individuals’ internal states can also affect portion size estimates, which relate to calories, with hungry participants displaying greater underestimation [48].

Since consumers are often inaccurate in estimating calories, they often resort to using other cues. In general, when consumers lack the relevant knowledge for evaluation of a particular product attribute, they may use other attributes that are seen as related to deduce product qualities [49,50,51]. This implies that various cues that are not necessarily intrinsically related to calories may affect caloric estimates.

Consumers may reduce calorie evaluations when they encounter product attributes that are seen as being correlated with low calories [52]. For example, low fat nutrition labels can lead to reduced calorie evaluations [53]. More generally, consumers underestimate calories in foods categorized as healthy [47,54,55]. Similarly, when consumers perceive a restaurant as healthier, they would tend to underestimate the calories in the dishes served in it [24,44,47]. Moreover, even product attributes that are unrelated to calorie content, such as being organic, can lead to reduced calorie estimates [52,56]. On the other hand, a smooth or soft food texture can lead to increased calorie estimations [57]. Such biases are all due to a natural association consumers have between healthiness and calories. 

While product attributes such as healthiness or low fat may have a reasonable bearing on calorie estimates, other characteristics that go beyond product attributes that bear less of an apparent link to calories have been shown to affect calorie estimation. For example, consumers tend to reduce their calorie evaluations of products marketed by companies with reputations for social responsibility or fair trade [58,59]. This alteration in calorie estimates occurs despite the fact that such attributes have no bearing on the actual calories contained in the product. 

In the current work, we suggest that visual cues, being extremely salient, may exert a strong influence on calorie estimates. When making food-related decisions, people tend to use salient cues more than subtle cues since salient cues are hard to avoid [18]. Research suggests that consumers tend to choose cues that are more salient or easier to process in product judgments [6,60]. This has particularly pronounced effects on judgment and decision making in the food domain. For example, food sound salience was found to increase people’s attention to the amount of food consumed and reduce consumption [19]. Additionally, presenting favorable caloric information on the front (vs. back) of a package enhanced consumption [20]. The more available a cue is, the more likely it is to be utilized in judgments of associated food product attributes. This in turn implies that visual cues, which are extremely salient, may have a sizeable impact on consumer judgment.

Next, we discuss the salience of visual cues and how this enhances their impact on consumer judgment.

### 2.2. The Importance and Influence of Images

Visuals, such as those given in product depictions and otherwise, serve as one of the most important communication tools of marketers [61,62,63], and they provide considerable information to consumers [14]. Visual cues have extensive effects on consumers’ attention, emotions, and behaviors [64,65,66,67]. Consumers are constantly exposed to multiple product visual depictions via various marketing platforms such as packages, print advertisements, video commercials, and billboards, as well as digital media in general and social media in particular [68,69,70]. Such venues present consumers with pictures of products, which may influence product evaluations. The prevalence of such cues makes them an important avenue of study. 

The strong influence of visual cues is partly due of their salience and ease of processing. In general, when making food-consumption decisions, people tend to grasp at available information, even if it is non-diagnostic [71]. Since consumers balance accessibility and diagnosticity, the accessibility of a piece of information may lead it to be utilized beyond what is merited by its diagnosticity [72]. Consumers’ preference for using more easily accessible cues [73,74,75] can help them conserve resources required for more elaborate processing. 

Visual elements are vivid and salient and can ease information processing [6]. Images, and particularly product imagery, tend to attract greater attention [76] and generate better recall than verbal claims [67]. Gathering visual information is intuitive, natural, and effortless for consumers [7]. This preference for accessible information may lead consumers to be heavily influenced by visual cues in product evaluation. Given their salience and ability to draw attention, visual inputs may have effects on product judgment that outweigh those of verbal claims [8]. The fact that visual information can be easily processed by consumers further contributes to their impact on product judgment and choice [76,77]. Next we discuss the specific effects visual inputs may have on consumer judgment.

### 2.3. The Effects of Visual Information on Consumer Experience and Judgment

Prior research has demonstrated extensive effects of visuals on consumer judgment and choice [78,79,80]. Product visual design influences consumers’ product impression, influencing product judgment on a variety of dimensions [76,79,81,82]. Product visuals serve as part of consumers’ overall product experience [83,84]. Specifically, visual cues induce consumer experience of emotions through non-instrumental product features such as shape, picture, and color [85,86]. Beyond general experience, visual elements may provide information concerning product attributes that cannot be directly experienced, such as smell or taste [76,87]. Accordingly, product images in particular can influence consumers on a variety of judgment dimensions [76,88,89].

The influence of visual cues on consumer judgment and choice is particularly pronounced in the food domain, where product visuals have a large impact on consumer judgment and choice [65,90,91,92]. Product visual design carries a variety of elements that influence a consumers’ initial impression of food products [93,94,95,96]. Typography can influence food product judgment [97], partly since it helps enhance readability [98] and facilitates consumer processing [63,99]. Depicting food products in motion can lead to increased evaluations of taste [100], healthiness [101], and overall appeal [102]. Specific image attributes such as shapes can even affect consumers’ attitudes [86] and sensory taste experience [96]. Finally, spokes-characters on children’s cereals can increase feelings of connection to the brand and choice [103] as well as enhancing the taste experience [104].

In sum, the salience and ease of processing of visual cues contribute to their extensive impact on food product evaluation and choice. Such impact may occur even when visuals convey no objective product information [8]. The specific effects of visual elements on product judgment may be due to the specific associations of these visual elements with product attributes, which we discuss next.

### 2.4. The Influence of Visuals through Associations with Product Attributes

Visual cues can be associated with product attributes in consumers’ minds and can therefore alter product evaluation on those attributes [105,106]. After an association between a visual element and a product attribute is established, consumers may alter their evaluation of the associated attributes when exposed to the associated visual cue. In this manner, undiagnostic cues can affect product judgment through learned associations, with the visual cue’s association serving as a heuristic for product evaluation [21]. For instance, repeated exposure to the co-occurrence of long product names with quality teaches consumers to associate name or description length with quality. Subsequently, consumers may infer quality from number of words [107].

Associations of visual images can affect evaluation even when the presence of the visual cues themselves is undiagnostic of actual product properties, merely due to learned associations. Indeed, the influence of visual elements on product judgment has been shown to extend to dimensions for which they are not relevant from an objective, informational perspective [21,108,109]. For example, kittens are associated with softness, such that pictures of fluffy kittens lead to judgment of toilet paper as softer [110]. In addition, presenting an image of a colorful sunset along with a brand resulted in brand beliefs that the product was more colorful [80]. 

Such effects of visual cues on associated attributes regardless of their actual diagnosticity have also been demonstrated specifically in the food domain. Names, fonts, shapes, size of containers, cartoon pictures, and colors can vary among different food products without any correlation with or direct claims regarding product attributes. Nonetheless, if these visual elements are associated with product attributes in consumers’ minds, they will affect the judgment of those associated attributes. 

For example, the formatting on a label may affect judgment of healthiness even when there is no difference in the information presented [111]. More specifically, a green color is associated with more environmental friendliness, such that using green visuals in advertisements can increase people’s perception of the brand’s environmental effort [112], further demonstrating the effects of visual cues through associations. Particular images can alter judgment of associated attributes. For example, displaying images of strong animals (e.g., a lion) on a pack of coffee can trigger a strength association, increasing judgment of coffee strength [113].

The current work focused on the association of size of image with product quantity and calorie estimation, which we discuss next.

### 2.5. Visual Cues and Calorie Estimation

Visual cues can specifically affect judgment of product quantity and calories [88]. When it comes to quantity estimates, product information, such as depicted portion sizes and explicit size labeling, has obvious and intuitively understandable effects on consumer quantity estimation. Thus, visual elements may constitute indirect cues regarding quantity through their association with quantity, and affect consumers’ estimation of serving size, quantity, and calories in products [114,115].

Visuals that on face value have no relation to objective quantity may affect judgment of quantity if they are associated with quantity in consumers’ minds. This is because, as explained above, the salience and ease of processing of visual attributes leads them to affect judgment of associated attributes. Different visual shapes may be associated with higher or lower quantity and may alter quantity estimates accordingly [116]. Similarly, different locations of graphics on packaging may be associated with volume and may accordingly alter judgment of product heaviness [117].

Visual elements may specifically alter estimation of calories in food products. Color can influence judgment of healthiness as well as calorie estimates [106]. Visual attributes such as the shape of a package can also influence both quantity and calorie estimation [114]. For example, front of product labeling, such as visually presented health claims, can influence calorie evaluation [118]. 

The impact of visual cues on calorie estimation is also explained by cue utilization theory [119]. This theory describes how people integrate information, which is embedded in various cues and received in diverse behavioral contexts in the formation of cognitive judgments [119,120]. Social psychology research has employed cue utilization theory to explain consumers’ inferential judgements [121] based on observable cues. Marketing research shows that cues, such as price and number of deals purchased, can affect product evaluation [122], online review helpfulness [123], and shoppers’ response to online promotions [124]. Accordingly, when product image size is available and easily accessible it may be taken as a relevant cue and utilized by individuals to form evaluations of product qualities, including caloric value.

In summary, visual cues may be influential in forming consumers’ estimations regarding food products’ quantity and calorie estimations. The salience and ease of accessibility of visual cues can promote their use in estimations of product calories when the cues are associated with calories in consumers’ minds. The issue with using visual cues is that they may not be diagnostic, and they may lead to biased quantity and calorie estimates [88,106,114].

### 2.6. Depicted Product Size and Calorie Estimate

When pictorial representations are larger, the product may be judged to contain more calories, even when the depiction does not in fact correspond to its actual size. This is because on an intuitive perceptive level, pictures are taken as their real-world counterparts [125]. Thus, a larger picture may be taken, by proxy, as indicative of a larger product.

Depicted package size is a primary cue for quantity estimation [126]. People learn to associate larger packages with greater product quantity, and so may infer greater quantity or calories from larger product packages [126]. Larger products typically imply greater product quantities, and therefore a greater number of calories. Similarly, larger pictures may also imply, by association, greater calories.

On a basic perceptual and motor level, people react to depicted objects as if they were real [127,128,129]. In the absence of the physical presence of a product, the size of product *depictions* may thus be taken as a real product, and consequently may serve as one prominent cue guiding product quantity evaluation. Given that pictorial representations do not exist in nature, the primitive human brain may take an image as an accurate representation for the actual object represented, taking its size as the size of the object represented even if they know it is not so on an explicit cognitive level. Consequently, the larger the product depiction, the larger consumers may intuitively perceive the product to be. Thus, they may deduce that the product possesses a greater number of calories.

In sum, given the salience of visual inputs and the association of size with quantity and calories on an intuitive level, larger product depictions should lead to increased estimates of quantity and calories. In other words, consumers may estimate greater calories when they encounter large product depictions. Accordingly, we predict the following:

**Hypotheses 1** **(H1).** *Larger size of product pictures will lead to increased calorie estimations*.

### 2.7. Picture Size May Influence Judgment of Calories Even When Objective Information Is Present

Given its ease of processing, visual information may be more influential in changing consumers’ perceptions of a food product than textual information, which requires more systematic type 2 processing [130]. Indeed, research has demonstrated the dominance of visual over verbal information in judgment, demonstrating that visual cues may override textual information and dominate it in determining product judgment [8]. Visual inputs also last longer in memory [131,132] and are generally better recalled than text [133]. The superiority of visual information occurs over other senses as well. For example, Schifferstein and Cleiren [134] have found that visual information dominates product experiences over other sensory experiences such as smell and sound. In sum, visual cues may override multiple other cues in affecting product judgment.

In the food domain, prior research has shown that visual cues determine product evaluation even in the presence of informative textual cues in the food domain [76,131,132,135,136,137]. Consumers may ignore objective information about food products, such as nutritional information, in their judgment [138], while being affected by visual cues, even if those are not as informative [11,91,139,140]. Given the salience and the strong impact of visual inputs on human judgment, the effects of picture size on product quantity estimation may occur even when consumers specifically possess objective information about product quantity. Visual cues associated with calories, such as the size of product image, may dominate over textual cues that are relevant to calorie estimation, such as information regarding product quantity.

Product quantity is highly relevant for the estimation of calories, since calories are a function of two key properties of a stimulus: (1) calorie density (i.e., type of food) and (2) portion size (i.e., quantity) [141]. Quantity is naturally associated with calories in this manner. Accordingly, research shows that the quantity of food displayed affects the perception of caloric content [43]. However, the dominance of visual cues may lead consumers to still bias their estimation of calories when shown visual cues associated with calories (i.e., increased product size in depiction), even when given more objectively relevant textual information regarding quantity. Relatedly, displaying calorie counts for drinks and deserts on restaurant menus did not lower calories ordered for drinks or desserts [142].

Accordingly, we predict that depicted product size may influence calorie estimates even when quantity information, which may objectively be more diagnostic of actual calories from a purely informative, rational perspective, is available. Formally:

**Hypotheses 2** **(H2).** *The effect on calorie estimation will be present even when consumers are given objective quantity information*.

We examined these hypotheses across two studies. Study 1 examined H1, demonstrating the effects of product picture size on calorie estimations, while study 2 examined H1 as well as H2, looking at the effects of picture size on calorie estimates in the presence of objective quantity information.

## 3. Study 1: Do Larger Package Sizes Lead to Increased Calorie Estimates?

Our first study aimed to examine whether the size of product pictures displayed to consumers influences calorie estimates. The study accomplished this by displaying different sizes of energy drink pictures to participants and examining whether or not this influenced their calorie estimates.

### 3.1. Method

#### 3.1.1. Participants 

Prolific academic workers (*N* = 130; 50% female; Age: *Mean* = 33.20, *SD* = 9.43) participated in the experiment for payment. This methodological approach was used in recent research that studied the effects of visual stimuli on consumers in general [143] and specifically in the food domain [101,144]. 

#### 3.1.2. Design and Manipulations

**Figure 1:**
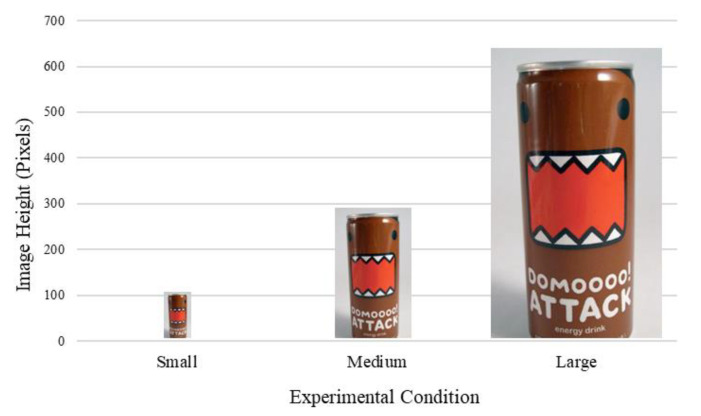
Product Picture Sizes Displayed to Participants (Study 1).

Participants completed the study online, signing up through the Prolific system. Participants were randomly assigned to one of three between-subjects conditions: small, medium, and large soda cans. Under each condition, they viewed pictures of an energy drink can modified to be presented in different sizes (102 vs. 288 vs. 640 pixels, respectively). The pictures, in the sizes displayed to participants, can be seen in Figure 1. Only visual and textual information were displayed on the can. No quantitative information regarding the drink was displayed (such as the quantity of the drink). Other than their size, the cans displayed were identical in each condition. Details other than the size of the can were varied between conditions.

Participants were shown a picture of a can of energy drink and were asked to estimate the number of calories in the can. Specifically, the instructions read: “Below is a picture of a bubble-gum flavored energy drink. Please have a look at it and answer the questions below.” Below the picture of the energy drink, participants read: “How many calories do you think a can of this drink has?” 

#### 3.1.3. Measures

Participants reported on the number of calories they estimated in their drink via an open measure. Answers were capped such that participants could not enter values greater than 1000.

### 3.2. Results and Discussion

We conducted an ANOVA model examining the effects of can size on estimated calories, with can size defined as the independent measure and estimated calories as the dependent measure. 

**Figure 2:**
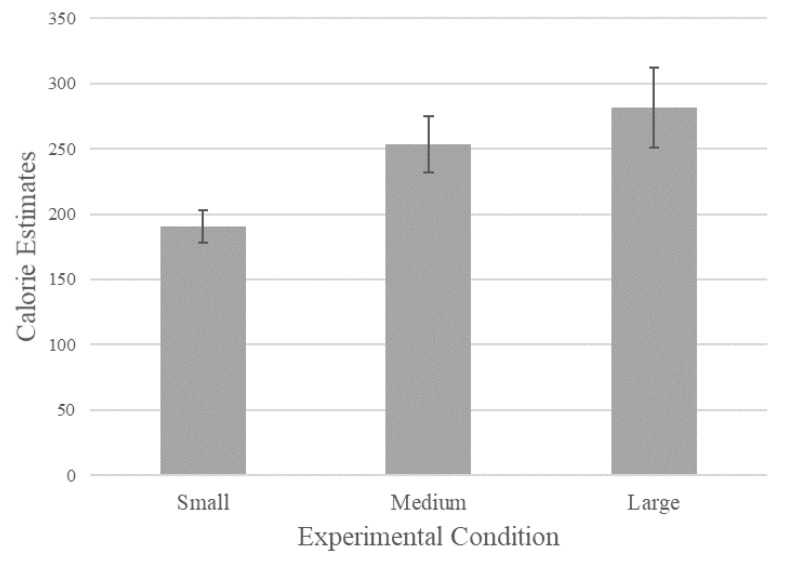
The Effect of Picture Size on Calorie Estimates (Study 1).

The size of the displayed soda can had a significant effect on estimated calories: *F*(2, 127) = 4.23, *p* = 0.02. Participants in the small can condition estimated an average of 190.59 calories (*SD* = 84.59, *n* = 44) in the small can condition, 253.67 calories in the medium can condition (*SD* = 140.28, *n* = 43), and 281.49 calories in the large can condition (*SD* = 201.33, *n* = 43). The results for calorie estimations in each experimental condition can be seen graphically in Figure 2. Full results can be seen in Table 1 below.

Planned contrasts revealed significant differences between the small and large can conditions, *F*(1, 127) = 8.05, *p* < 0.01, and between the small and medium conditions, *F*(1, 127) = 3.87, *p* = 0.05. The difference between the medium and large conditions was not significant, *p* > 0.10.

Overall, these findings suggest that the size of product pictures displayed to consumers influenced calorie estimates, supporting H1. Specifically, larger product pictures led to increases in the number of calories estimated by consumers. Next, we aimed to examine our second hypotheses to see whether or not visual cues override relevant information provided by text. 

## 4. Study 2: Are Larger Package Graphics More Influential Than Objective Information?

The second study aimed to examine whether visual size cues influence calorie estimates even when quantity is objectively known. The study used the same general paradigm used in the previous study, while providing objective quantity information, to examine whether participants would still be influenced by product picture size in their estimation of calories in the product, consistent with H2. Product quantity is objectively related to calories, given that calories in a product are a function of product quantity and calorie density. However, if visual cues indeed supersede objective information in arriving at calorie estimates, size of product picture should still influence calorie evaluation, even given objective information regarding product quantity.

### 4.1. Methods

#### 4.1.1. Participants and Setting

Seventy-three undergraduate students (62% female; Age: *Mean* = 24.45, *SD* = 7.67) were recruited from the participant pool of a large Northeastern university. They completed the study for payment or credit. The study was conducted in the behavioral lab of the college. The study was completed as a printed questionnaire, using pen and paper.

#### 4.1.2. Design and Manipulations

Participants were randomly assigned to one of three between-subjects conditions (small soda can, medium soda can, large soda can). Participants were shown a picture of a can of energy drink and were told the number of ounces that the soda can held. The pictures were similar to those used in the previous study. The quantity of drink contained in the can was emphasized at the top of the instructions page, above the product picture. While the reported quantity remained the same for all participants, the pictured size of the soda can varied between participants. Specifically, participants read: “Below is a picture of a bubble-gum flavored energy drink. Have a look at it and answer the questions below. The can contains 8.4 ounces (250 mL).” (Emphasis in original).

#### 4.1.3. Measures

Participants were asked to estimate the caloric content of the soda can. They estimated this on a simple open question asking how many calories the can had. We also measured how many ounces participants thought the soda cans contained. Recall that participants were told how many ounces the soda can contained on page 1 of the questionnaire. They were then asked to report the number of ounces contained on page 2 and were instructed not to look back at the quantity stated on the first page. Since participants were told how many ounces each product contained, this was intended as a manipulation check, and allowed us to examine distortion of quantity based on caloric density and whether people can exaggerate calorie estimates even when they know the objective quantity a product contains. In this, the study examined the dominance of biasing visual cues that affect calorie estimations over objectively relevant nutritional information.

### 4.2. Results and Discussion

**Figure 3:**
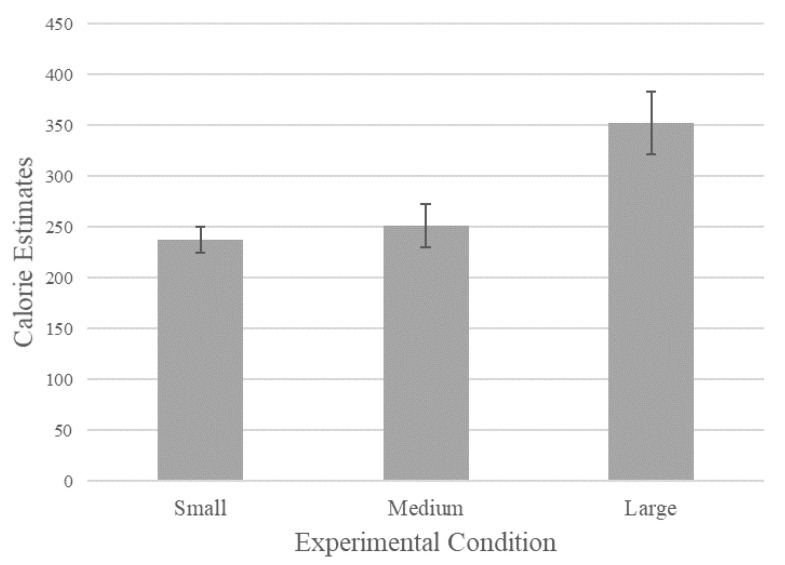
The Effect of Picture Size on Calorie Estimates (Study 2).

Two outliers were eliminated due to a number of calorie estimations 3 standard deviations above the mean (estimations of 800 and 1000 calories). An ANOVA model of the effects of depicted can size (small vs. medium vs. large pictured can) on perceived calories revealed the predicted effect: soda can size had a significant effect on calorie estimation (*F*(2, 68) = 4.7; *p* < 0.01). Participants in the small-pictured soda can condition judged the soda can as containing fewer calories (*M* = 237.67, *SD* = 89.52, *n* = 24) than those in the medium-pictured soda can condition (*M* = 251.74, *SD* = 154.77, *n* = 23). Those in the medium-pictured soda can condition perceived the soda can as containing fewer calories than those in the large-pictured soda can condition (M = 352.27, SD = 158.14, *n* = 22). The results for calorie estimations in each experimental condition can be seen graphically in Figure 3. Without excluding outliers, results displayed an even stronger pattern, 237.67 (89.52) for small, 343.6 (391.21) for medium, and 431.25 (319.77) for large. 

We conducted planned contrasts to examine differences within the particular conditions. Doing this, we found differences between most of the conditions. There was a significant difference between the medium and large cans, *F*(1, 68) = 6.07, *p* = 0.02, and between the small and large cans, *F*(1, 68) = 8.05, *p* < 0.01. The difference between the small and medium cans was not significant, *p* > 0.10.

We also measured how many ounces participants thought the cans contained (there were no significant differences between conditions, *p* = 0.65). Controlling for recalled quantity via ANCOVA, including it as a covariate, had no effect on estimated calories. The effects of picture size on calorie estimates were still significant at the 0.01 level, *F*(2, 57) = 4.48.

It appears that consumers took the size of picture as a cue to calories contained in the can. Since calories are related to size, consumers estimated a higher number of calories from the increased size of graphic elements. The bigger the product depiction, the more calories they estimated the can had. 

Notably, size of picture led to inflation of calories *regardless* of the quantity information, even though in this case the quantity was displayed on the can. To ensure quantity was indeed known, we controlled for estimated weight in the above analysis, using estimated weight as a covariate. We also ran an ANOVA of the effects of picture size on estimated weight, finding no effects (*p* = 0.64): participants viewing different picture sizes estimated similar quantities in ounces, unsurprising given that most participants correctly recalled weight and that energy drink cans typically contain fairly standardized quantities. This makes the calorie finding particularly remarkable—participants estimated a higher quantity of calories even though they correctly remembered equal amounts of soda, despite the strong objective connection between quantity and calories. 

Graphic elements appear to be interpreted as such a robust cue of dimensions such as product weight and calories that even when particular quantity elements are known, consumers may distort their estimates of such variables. Thus, increased size of graphic elements led to increased quantity judgment (in this case, calories).

## 5. General Discussion

### 5.1. Discussion

The studies presented here demonstrate that consumers take non-diagnostic visual cues as being indicative of conceptually associated but unrelated food product attributes. Specifically, we have shown that the size of a product picture influences estimated calories. Furthermore, we demonstrated that consumers are biased by variation in product image size even when in possession of more diagnostic but textual (rather than visual) information regarding product quantity. This constitutes a novel and to our knowledge previously undocumented biasing effect in calorie estimation. Prior research has not to our knowledge documented effects of visual product representations on calorie estimates. Moreover, the study provides, to our knowledge, a first direct demonstration of the way visual cues can override relevant textual cues (quantity) regarding calories.

The effects of visual cues on nutrition estimates likely occurs because of the salience and ease of processing of visual cues, which leads it to be utilized over more diagnostic but less salient and easy to process information (such as deducing calories from quantity). Such cues provide vast and important information to consumers [14] and have broad effects on consumers’ attention, emotions, and behaviors [64,65,66,67]. This may explain why visuals, which are frequently utilized in advertisements, product packaging, and various media, constitute one of the major communication tools of marketers [61,62,63].

Visual cues can be associated with product features in consumers’ minds and can thus be utilized in product estimation of these features [105,106]. Therefore, undiagnostic cues can effect product perception through learned associations, with the visual cues’ association serving as a heuristic in product assessment [21]. Accordingly, our findings strengthen previous research [88,106,114] by suggesting that such use of undiagnostic visual cues may lead to biased quantity and calorie estimates.

Consumers learn to associate the size of product depictions with calories, given that larger packages typically carry greater amounts of food. This association of large visual size of packages with quantity and calories may generalize to product pictures, leading larger pictures to increase judgment of food quantity and calories.

Importantly, picture size influenced quantity judgment even when product quantity was known (study 2). Consumers seemed to give significantly less weight (or even to ignore) diagnostic information in favor of salient and easy-to-process, yet non-diagnostic, cues. These findings are in line with studies that demonstrated that visual inputs, in particular, may offer a salient cue to guide calorie estimates and override textual cues in guiding consumer judgment and decision making [6,7,8].

### 5.2. Contributions and Implications

The current research offers a novel finding regarding the influence of pictorial size cues on calorie estimates, as well as providing direct evidence for the dominance of visual cues over textual information in calorie estimates. The findings are important because food quantity estimates have a major role in shaping consumers’ food choices and consumption quantity [22,23]. Overall, people tend to increase consumption of food products that are perceived to be less caloric and lower in quantity [22,24]. This may potentially lead to overconsumption, and consequently may contribute to obesity [13,29,30]. Generally, consumers tend to underestimate calories, which may lead to overeating [33]. Overeating, in turn, may lead to extensive adverse health effects and concomitant social costs [34,35,36]. Therefore, it is important for both researchers and practitioners to explore, reveal, and understand the factors that bias calorie estimation.

The studies contribute to our understanding of how consumers form calorie estimations, indicating that they may utilize easily available elements from their environment in their judgment of calories, and potentially other important product attributes. The studies also contribute to our understanding of how consumers estimate calories, providing further testament to the malleable nature of such estimates. In addition, the studies add to our knowledge concerning how visual elements affect not just overall product evaluation but also judgment of specific product attributes.

These findings have important implications in the marketplace. Specifically, in restaurant and grocery store settings where consumers decide on products based on package sizes or labels, misattributions of this kind can be especially influential and misleading to consumers, leading to mistakes in estimated calories. While it is sensible that elements such as bag size (which may not in itself be indicative of product quantity) affect quantity and calorie estimations, the current study demonstrates that size elements bearing no direct relation to quantity can also influence quantity judgment. This emphasizes the importance of easily available calorie information on packaging.

The findings are also important from the perspective of consumer welfare, as they reveal that visual information can bias consumers from the objective information required by regulators. Consumers with low nutritional literacy may be particularly vulnerable to these biasing effects. Hence, it is important to understand what extraneous factors may bias consumers’ nutrition assessment in order to help protect consumers from undue bias. 

Our research also has important implications for consumer health. In an attempt to maintain congruity and conserve resources by implementing accessible cues, consumers may conflate aspects of product presentation with characteristics of the food itself. This leaves consumers vulnerable to being misled. Research on health halos suggests that emphasizing the healthfulness of a meal can promote overeating and, paradoxically, unhealthy eating behaviors [145]. Thus, understanding biases such as the present one is important in preventing biases and providing diagnostic product information that is both more accessible and easier to process, which can ultimately help guide healthier eating.

Due to consumers’ willingness to forego diagnostic information in favor of being influenced by associations of highly available cues such as visuals, understanding the implications of highly accessible cues such as visuals may be of high importance to consumer welfare. Stable associations between desirable product attributes and indirect cues may adversely affect consumer decision making by biasing product judgment. This may be especially true given that indirect cues to product attributes are less likely to generate consumer counter argumentation and discounting. Accordingly, indirect cues to product attributes (e.g., larger visuals) may have a greater effect on consumers than direct claims (e.g., this product is high calorie) [136,137].

Finally, if inflated estimations of product calories does indeed have downstream effects on consumed judgment, the findings may suggest a useful intervention, whereby leading people to overestimate the calories of a product can assist them in eating less, under the illusion of having eaten more, which may contribute to psychological satiety. In this manner, biasing consumers to believe there are more calories in a product may actually work in their favor by leading them to regulate their consumption more carefully and consume lower amounts.

### 5.3. Limitations and Future Research

The current studies demonstrate the effects of product picture size on calorie estimates. Further studies may explore the effects of other visual inputs that may be associated with calorie estimates, such as the size of graphic elements on the package. For example, various elements of font design—font height, spacing between letters, letter bulkiness, etc., may affect calorie estimates. Similarly, depictions of the product on the packaging, showing larger or smaller product depictions (e.g., the size of cereal pieces) or larger or smaller quantity (e.g., in a cereal bowl) may influence estimates of product quantity or calories.

Studies may also explore additional cues to quantity the impact of a package’s graphic properties. For example, the space between the logo and the edges of a package may hint at quantity in the same way that spaces between the food and the plate on which it rests affect calorie judgment [138]. Furthermore, the context in which a product is displayed—for example shelf size and height—may influence calorie judgment, either via assimilation or contrast with its environment, as in a context’s effect on size judgment, for example with the Ames room [139,140].

In the current research, both studies demonstrated the effects of product picture size on calorie estimates. Study 2 demonstrated that visual size cues influence calorie estimates even when quantity is objectively known. This was done by manipulating only the size of the image presented to participants. Future research could include a control product to examine the relative impact of the different cues presented.

Further research should also examine the process involved in producing the effects of product depiction size on calorie estimates. As discussed above, we suggest that the accessibility of visual size cues contributes to their influence on calorie judgments and that people take such elements as a heuristic cue to quantity. If this is indeed the case, we should see increased effects of such cues under cognitive load, a proposition that can be tested in further studies. Moreover, providing other accessible cues to quantity or making other cues, such as nutritional information, easier to process, should mitigate the effects of visual depiction size on calorie estimates.

Further studies can also investigate the role of consumers’ nutritional literacy in susceptibility to biases in calorie estimates generated by visual cues. It may be interesting to investigate whether or not consumers with higher nutritional literacy are less susceptible to biases generated by visual information when being given objectively relevant information, such as quantity.

The role of salience can also be explored by pointing consumers’ attention towards or away from graphic elements and seeing whether such measures moderate the effects. This would also clarify the role of conscious attention in generating our effects. If rendering visual cues less salient reduces their effect, this would support the role of the salience of such clues in generating effects. Similarly, if rendering visual cues in a way that is easier to process reduces their effects, this would support the role of ease of processing in generating the effects of size cues on caloric estimates.

The effects demonstrated in the current work may partly be driven by a need for consistency, seeing quantity as consistent with size. Seeing product calories as being consistent with other size-related elements is less cognitively demanding and may be more emotionally satisfying due to cognitive consistency needs than seeing product depiction size as being inconsistent with calories. This proposition can be tested in further studies controlling for the need for consistency and demonstrating the involvement of ease of processing and positive affect that is due to consistency in the process of attributing quantity to products with large product depictions.

The involvement of the association between a products’ visual size and calories can also be investigated by exposing consumers to products where such association is strengthened or reduced and examining the effects of such exposure on subsequent quantity judgments. Teaching consumers that the size of visual inputs is or is not tied to product quantity may moderate our effects, providing potential avenues for debiasing. 

Given that product pictures can be displayed in different sizes in different contexts, the findings may generalize to various settings where people are exposed to product pictures. For example, seeing larger or smaller street signs depicting the product may affect consumer judgment of quantity or calories. Similarly, larger or smaller displays in fast food restaurants may also bias quantity estimates. Finally, the size of visual cues on product packaging, such as depictions of the product itself, the size of its logo, or depicted serving size, may also affect calorie estimates. Future research should examine whether the effects indeed generalize to these, and other, ecologically and practically relevant settings. 

A variety of other implicit associations between package attributes and conceptually aligned product attributes may play a role in how food manufacturers design their packages and other visual marketing communication. While size and quantity have direct and obvious effects on consumer well-being, other interesting pairings of package attributes and product associations may be explored. Recognizing the association consumers have formed between the color green and healthfulness, for example, package designers may place unhealthy foods into green packages, misleading consumers into believing that they are making healthy food choices [106]. Claims that speak to the environmental friendliness of a package (e.g., “printed on recycled cardboard”) may mislead consumers to believe that this show of environmental consciousness will translate into the health consciousness of the food item. The use of more elegant or explanatory packaging may tap into consumer’s beliefs that more expensive food items are healthier [141]. The sturdiness of a product box or package may lead consumers to rate the product itself as being more durable. Package color, such as red and blue, may cue and affect perceptions of product temperature or spiciness. A food or beverage product packaged with a slender design may be rated as more likely to make the consumer slender. Further research may explore additional effects of visual design elements on judgment of associated product attributes.

Further studies can also explore downstream consequences of the alterations in calories estimates displayed in our studies. Specifically, studies can investigate whether such increased estimations of calories lead to corresponding decreases in purchase or influence consumption quantity. If a person thinks that a product has a greater or lesser quantity or that it is more or less caloric, they may consequently consume less of the product [24,142,143,144,145]. Future research can help examine ways to minimize visually generated biases in consumer nutritional assessment. Such research can help regulators protect consumers, particularly ones with low nutritional literacy, who may be more susceptible to biases, from the biasing effects of irrelevant visual cues.

## 6. Conclusions

Consumers face different types and forms of information when making product choices. Among other aspects, they may consider the product’s nutritional properties. The current article shows that visual cues may exert a strong influence on consumers’ nutritional assessments, biasing their evaluation of nutritional information. Specifically, seeing larger product depictions lead to increased calorie estimates. More generally, highly available cues may be utilized by consumers in evaluating food products, above and beyond the “appropriate”, objective information.

Currently, explicit product claims are regulated, but factors that indirectly affect consumer judgment without making direct claims remain unregulated. This is despite their great impact and ability to distort consumer perception, as suggested by the current research. This work shows that companies may employ elements that do not directly present product attributes, such as product depictions, in a manner that biases product judgment. Accordingly, it stresses the importance of noting potential biasing visual cues given by marketers, indicating a need for awareness of these biases by marketers, consumers, and regulators alike.

## Figures and Tables

**Table 1:** The Effect of Picture Size on Calorie Estimates.

Condition	N	Mean	SD	Min	Max
Small	44	190.59	84.59	5	350
Medium	43	253.67	140.28	100	800
Large	43	281.49	201.33	40	1000

## Data Availability

The data that support the findings of this study are available from the corresponding author upon reasonable request.
